# *Ulmus parvifolia* Accelerates Skin Wound Healing by Regulating the Expression of MMPs and TGF-β

**DOI:** 10.3390/jcm9010059

**Published:** 2019-12-26

**Authors:** Min Cheol Kang, Silvia Yumnam, Woo Sung Park, Hae Min So, Ki Hyun Kim, Meong Cheol Shin, Mi-Jeong Ahn, Sun Yeou Kim

**Affiliations:** 1 College of Pharmacy, Gachon University 191, Hambakmoero, Yeonsu-gu, Incheon 21936, Korea; mincjf07@gmail.com (M.C.K.); silviayumnam@gmail.com (S.Y.); 2 College of Pharmacy and Research Institute of Pharmaceutical Sciences, Gyeongsang National University, Jinju 52828, Korea; pws8822@gmail.com (W.S.P.); shinmc@gnu.ac.kr (M.C.S.); mjahn07@gmail.com (M.-J.A.); 3 School of Pharmacy, Sungkyunkwan University, Suwon 16419, Korea; haemi9312@naver.com (H.M.S.); khkim83@skku.edu (K.H.K.); 4 Gachon Institute of Pharmaceutical Science, Gachon University, Yeonsu-gu, Incheon 21936, Korea

**Keywords:** *Ulmus parvifolia*, wound healing, matrix metalloproteinase, transforming growth factor, skin rejuvenation

## Abstract

*Ulmus parvifolia* is one of the medicinal plants used traditionally for treatment of wounds. We intended to investigate the wound healing effect of the powder of *Ulmus parvifolia* (UP) root bark in a mouse wound healing model. We also determined the mechanisms of effects of *U. parvifolia* in skin and skin wound healing effects using a keratinocyte model. Animal experiments showed that the wound lesions in the mice decreased with 200 mesh *U. parvifolia* root bark powder and were significantly reduced with treatment by UP, compared with those treated with *Ulmus macrocarpa* (UM). Results from in vitro experiments also revealed that UP extract promoted the migration of human skin keratinocytes. UP powder treatment upregulated the expression of the matrix metalloproteinase-2 and -9 protein and significantly increased transforming growth factor (TGF)-β levels. We confirmed that topical administration of the bark powder exerted a significant effect on skin wound healing by upregulating the expression of MMP and transforming growth factor-β. Our study suggests that *U. parvifolia* may be a potential candidate for skin wound healing including epidermal skin rejuvenation.

## 1. Introduction

Skin is composed of the dermis and epidermis layers. Skin protects our body against environmental factors such as harmful ultraviolet (UV) rays and pathogens and prevents water loss from the body [1]. When skin is injured, skin repairs itself. The wound healing process in a complex multistep which includes blood clot formation, wound inflammation, and skin tissue proliferation and remodeling [2]. Wounds begin to heal immediately after an injury to release various clotting factors. During the inflammatory phase, neutrophils and macrophages are activated by the release of proinflammatory cytokines including Interleukin (IL)-1𝛽, IL-6, IL-8, Tumor necrosis factor (TNF)-α, and growth factors such as platelet-derived factors (PDGF), transforming growth factors (TGF), Insulin-like growth factor (IGF)-1, and fibroblast growth factors (FGF). In the proliferative phase, these factors stimulate proliferation and migration of cells to move to the injured site for extracellular matrix (ECM) formation. Finally, fibroblast and vascular density decrease during the remodeling phase, old collagen fibers of the initial scar are replaced with matrix, and new collagen fibers are synthesized to form new tissue [3,4,5,6].

Because the skin healing process is very complex, there can be limits to fully overcoming wound injury with a single compound. Thus, the development of wound healing agents with natural products may be an option for cutaneous wound treatment. The use of natural products as wound healing materials can have some advantage such as low cost and high safety in comparison to other synthetic agents. For thousands of years, many natural resources have been reported to be used for skin injury. Over the last 10 years, many studies have reported evidence that natural products can improve skin wounds [7]. As part of such research, this study was conducted to demonstrate the pharmacological function of elm tree for skin wounds.

The elm tree is widely distributed in Asia and its stem and root barks have use in traditional oriental medicine to treat gastric disorders and intestinal inflammation [8]. Particularly, it has long been used in regenerating stomach or skin epithelial cells. Korea elms are also known for their effects on blood circulation, the protection of cartilage degeneration, and damaged tissue regeneration [9]. With regard to topical use, elms have been administered for the treatment of minor skin irritations, cold sores, ulcers, abscesses, and boils [10]. Bioactivities of various Ulmus species have been reported. *Ulmus davidiana* var. *japonica* has antioxidant, anti-inflammatory, and immune-modulating effects [11]. Recent studies on *U. parvifolia* Jacq. (UP), a species of elm native to China, Korea, and Japan, have shown that its leaves and stems have anti-inflammatory and antioxidant effects [12]. *U. parvifolia* bark, which contains phenolic compounds and steroidal glucosides, is used for the treatment of eczema and edema [13]. Water-soluble extracts of the root bark of *U. parvifolia* showed anti-inflammatory properties, and cotreatment with the mycelia of mushroom protected against allergic asthma in mice [14,15]. Interestingly, the powder of the original *U. parvifolia* material itself has also been used in oriental medicine, rather than being used only as an extract for a clinical purpose. Therefore, our study aimed to check the possibility of *U. parvifolia* as a candidate for skin wound healing. Firstly, we investigated the influence of dorsal treatment with *U. parvifolia* in the animal model of cutaneous wounds according to the particle size of the *U. parvifolia* root bark power. Furthermore, we performed a comparative study of species differences, such as *U. parvifolia* and *U. macrocarpa*, on potential efficacy in skin wound models.

## 2. Materials and Methods

### 2.1. Sample Preparation

*Ulmus parvifolia* was collected from Busan and Jinju, provided by Prof. MJ Ahn at Gyeongsang National University, Jinju 52828, Korea, in April 2018. The root barks were washed with water, dried, and pulverized using a grinder. Each powder was sieved through 20, 50, 100, and 200 mesh sieves (pore sizes: 0.85, 0.35, 0.15, and 0.075 mm, respectively), to obtain 4 grades of root bark powder. UP powder was extracted twice in 80% methanol for 24 h with 1 h sonication. The solution was filtered through Whatman No. 1 filter paper (GE Healthcare, Cleveland, OH, USA), concentrated using a rotary vacuum evaporator under reduced pressure. The extract was dissolved in dimethyl sulfoxide (DMSO) for in vitro use.

### 2.2. Measurement of the Angle of Repose of the Powder

The angle of repose (θ) for the root bark powder was measured using the cone height method. Briefly, a funnel was fixed at a height of 30 cm (H) above ground level, and different sizes of the powder were allowed to gently flow through it until the tip of the powder cone touched the outlet of the funnel. The diameter (2R) of the cone was measured for each powder type. The angle of repose (θ) was calculated as follows:

θ = tan − 1 × (h/r)(1)

This test was performed in triplicate for each sample.

### 2.3. Wound Healing Model

Specific, pathogen-free, 5-week-old male SKH-1 hairless mice were purchased (Orient Bio; Gyeonggi-do, Korea) and acclimatized for 1 week in a temperature- and humidity-controlled room (23 °C and 60% humidity), under a 12 h light–dark cycle, before the start of the experiments. All experimental protocol for animal experiments was reviewed and approved by the animal care committee of the Center of Animal Care and Use (CACU, LCDI-2018-0007) at the Lee Gil Ya Cancer and Diabetes Institute, Gachon University, Korea. Set A: The mice were randomly divided into 5 groups (*n* = 7). The mice were anesthetized using 5% isoflurane, and the skin was cleaned with 70% ethanol. Two excision wounds were created in the posterior dorsal area of each mouse using a 6 mm biopsy punch (0.28 cm^2^), Each wound was (1) untreated; or (2) treated with 50 mesh (12 mg); (3) 100 mesh (12 mg); or (4) 200 mesh (12 mg) root bark powder of UP; and (5) Madecassol^®^ (12 mg, positive control, Dongkook Co.; Korea) topically applied. The wounds were covered with a commercial dressing Tegaderm (3M) to prevent wound infection. The wound was treated once daily for 6 days until the day of sacrifice. Set B (large scale wounds): The mice were randomly divided into 4 groups (*n* = 8). The mice were anesthetized using 5% isoflurane, and the skin was cleaned with 70% ethanol. An excision wound was created on the dorsal by cutting out a circular region, 20 mm in diameter, with surgical scissors. Each wound was (1) untreated; or (2) treated with UP 200 mesh (20 mg); (3) *U. macrocarpa* (UM) 200 mesh (20 mg); and (4) Madecassol^®^ (20 mg, positive control) topically applied. The wounds were covered with a commercial dressing (Tegaderm, 3M, MN, USA) to prevent wound infection. The wound was treated once daily for 15 days until the day of sacrifice.

### 2.4. Wound Analysis and Histological Assessment

Digital photographs of the wounds were captured on each day of treatment or at day 0, 3, 7, 10, and 14, using a digital camera (Olympus, Tokyo, Japan), and ImageJ software (version 1.5a; Bethesda, MD, USA) was used to measure the wound sizes. Mice were sacrificed at the end of experiments after grafting for histological assessment. The harvested wound areas, including a border of normal tissue, were immediately fixed in 10% neutral-buffered formalin. The specimens were embedded in paraffin, sectioned, and stained with hematoxylin and eosin (H and E) and Masson’s trichrome (MT). ImageJ software (version 1.5a) was used for the quantification of collagen in tissue sections.

### 2.5. Western Blotting

The harvested skin tissues were homogenized in Pro-prep solution (iNtRON Biotechnology; Seoul, Korea), and their lysates were centrifuged at 12,000× *g* for 30 min. The proteins were separated by SDS-PAGE and transferred onto a polyvinylidene difluoride (PVDF) membrane (Millipore, MA, USA). The membranes were blocked with 5% nonfat milk for 2 h and washed with Tris-Buffered Saline containing 0.05% Tween-20 (TBST) buffer. The membranes were incubated with primary antibodies of Matrix metalloproteinase (MMP)-1 (ab137332, 1:1000, abcam, Cambridge, UK) -2, -9, and TGF-β1 (sc-13595, sc-393859, sc-130348, 1:1000, Santa Cruz, CA, USA), at 4 °C overnight. The blots were incubated with a horseradish peroxidase-conjugated secondary antibody (1:1000, Thermo Scientific, IL, USA) for 1 h. Immunoreactive bands were visualized with the Pierce ECL Western blotting substrate (Thermo Scientific, IL, USA), using ChemiDoc (BioRad Laboratories, CA, USA).

### 2.6. Cell Culture

HaCaT cells were obtained from the Korean Cell Line Bank (Seoul, Korea). The cells were cultured in high-glucose Dulbecco’s modified Eagle’s medium supplemented with 10% fetal bovine serum (FBS, Gibco, NY, USA) and 1% penicillin–streptomycin (WelGENE, Daegu, Korea) in 5% CO_2_ at 37 °C.

### 2.7. Cell Viability Assay

The cytotoxicity of UP extract was examined using the 3-(4, 5-dimethylthiazol-2-yl)-2,5-diphenyltetrazolium bromide (MTT, Sigma, MO, USA) assay. HaCaT cells were seeded into 96-well plates (4.0 × 10^4^ cells/well) in 10% FBS-containing medium. The cells were treated with various concentrations of UP extract, diluted in serum-free media. After 24 h of incubation, 0.5 mg/mL MTT solution was added, and the cells were cultured for 1 h. The dark-blue formazan crystals were solubilized with dimethyl sulfoxide (DMSO, Sigma, MO, USA), and the absorbance at 570 nm was measured using a spectrophotometer (Molecular Devices, CA, USA).

### 2.8. Cell Migration Assay

HaCaT cells were seeded in 96-well plates (3.0 × 10^4^ cells/well) for the scratching assay. Monolayers of cultured cells were subjected to scratch wounds with a Wound Maker tool (Essen Bioscience, MI, USA), and the media was removed by suction. The cells were then washed twice with PBS buffer and incubated for 12 h in the presence or absence of UP extract. IncuCyte ZOOM (Essen Bioscience, MI, USA) was used to inspect cultures every 2 h.

### 2.9. Statistical Analysis

Differences between groups were determined using a one-way analysis of variance (ANOVA). *p*-values of <0.05, <0.01, and <0.001 were considered statistically significant. Results are presented as the mean and the standard error of the mean (SEM).

## 3. Results

### 3.1. The Angle of Repose of Different Particle Sizes of the Root Bark Powder of U. parvifolia

The angle of repose indicates changes in the fluidity in the root bark of UP. The angle of repose for the different particle sizes of the root bark powder of UP is shown in Table 1. The root bark powder of UP with a particle size of 200 mesh (49.8 ± 1.1°) had a lower angle of repose than the others, followed by 100 mesh (51.9 ± 1.6°) and 50 mesh (52.9 ± 0.7°), with the highest being the 20 mesh (57.2 ± 0.8°). The angle of repose of the root bark powder decreased as the particle size decreased

### 3.2. Effect of the Particle Size of the Root Bark Powder of U. parvifolia on Wound Healing in Mice

**Figure 1:**
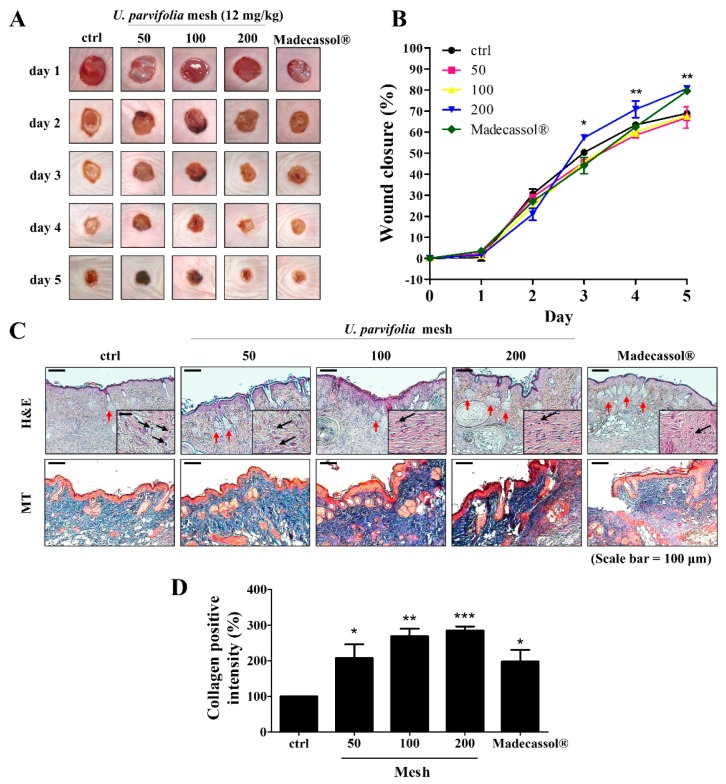
Effects of the root bark of *U. parvifolia* on wound healing in hairless mice. (**A**) Representative images of wounds from each group over a five-day period post-wounding. Madecassol^®^ was used as positive control. (**B**) The graphical representation of the average wound closure in each group was measured using ImageJ software. (**C**) H and E-stained skin tissue sections and Masson’s trichrome-stained sections on day 5. Scale bar = 200 μm. Black arrow indicates inflammatory cells and red arrow indicates the hair follicle and glands in the wound site. Insets of main figures represent granulation tissue (50 μm). (**D**) Graphical representation of expression of collagen formation in dorsal. The values are shown as mean ± SEM (*n* = 7). * *p* < 0.05, ** *p* < 0.01, and *** *p* < 0.001 vs. the control group.

We observed the regenerative effects of the root bark powder of UP using a SKH-1 hairless mouse model. To assess the efficacy of UP powder, wound closure was observed after treatment with UP powder (50, 100, and 200 mesh) for five days. Wounds treated with the 200 mesh powder showed a faster rate of wound closure and dermal regeneration compared with those treated with other sizes (Figure 1A). In the 200 mesh treatment group, wound sizes were significantly decreased on day 5, whereas those in the control group were not healed (Figure 1B). In addition, we investigated the tissue samples of skin wounds using H and E and MT staining. Treatment with 200 mesh UP powder resulted in increased granulation tissue formation, hair follicle, and glands, and decreasing of inflammatory cells in the epidermis was found compared with that in the untreated group (Figure 1C). Collagen formation was significantly increased in the 200 mesh treatment group (Figure 1D).

### 3.3. Effects of Root Bark Extract of U. parvifolia on Migration in HaCaT Cells

**Figure 2:**
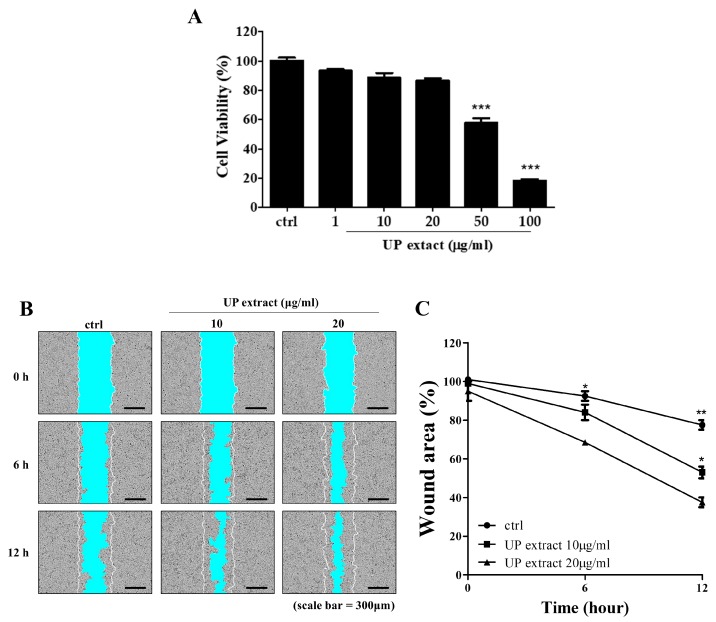
Effects of UP extract on migration in HaCaT cells. (**A**) Cells were cultured in 96-well plates and treated with UP extract (1, 10, 20, 50, and 100 μg/mL). After 24 h, cell viability was measured using the MTT assay. (**B**,**C**) Wound areas were recorded over time using the IncuCyte ZOOM™ live cell-imaging platform. HaCaT cells were cultured with or without UP extract. The red line indicates the initial scratch wound mask, created immediately after wound creation. The values are shown as mean ± SD (*n* = 6). * *p* < 0.05, ** *p* < 0.01, and *** *p* < 0.001 vs. the control group.

To determine whether root bark extract of UP affected rejuvenation and wound repair, we induced wounds in skin keratinocyte (HaCaT cells) monolayer cultures and administered the root bark extract of UP. As shown in Figure 2A, no significant change in cell viability was noticed after treatment with root bark extract at 10 μg/mL. HaCaT cells grown in the presence of root bark extract of UP showed faster, dose-dependent growth rates compared with the untreated cells (Figure 2B,C).

### 3.4. Effect of the Root Bark Powder of U. parvifolia on Large-Scale Wound Healing in Mice

**Figure 3:**
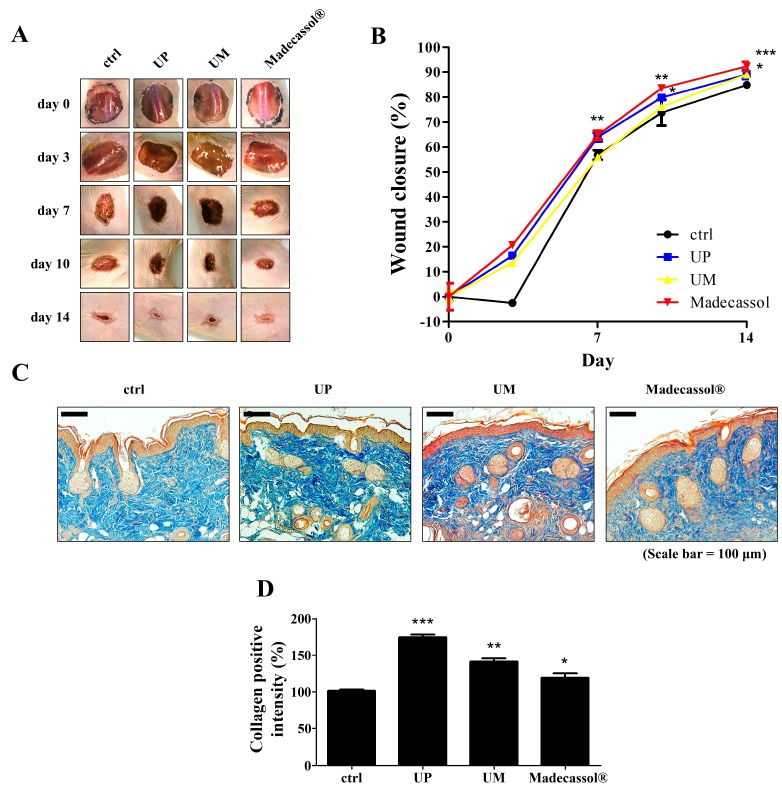
Effects of the root bark of *U. parvifolia* and *U. macrocarpa* on wound healing in hairless mice. (**A**) Two Ulmus root barks were applied to the wounds of SKH-1 mice for 14 days. Madecassol^®^ was used as positive control. (**B**) The closure rates of 20 mm diameter wounds were measured. (**C**) Masson’s trichrome-stained tissue sections on day 14. Scale bar = 100 μm. (**D**) Graphical representation of expression of collagen formation in dorsal. The values are shown as mean ± SEM (*n* = 7). * *p* < 0.05, ** *p* < 0.01, and *** *p* < 0.001 vs. the control group.

To investigate the effect of the root bark powder (200 mesh) of UP in large-scale wound healing, we observed its regenerative effects using a 20 mm diameter wound created on SKH-1 mice. We also treated the wound with the root bark powders of UM to compare the effects of these with those of UP powder. Wounds treated with UP powder showed a faster rate of wound closure and dermal regeneration, similar to treatment with Madecassol^®^ powder, 7 and 14 days post wound creation (Figure 3A). Seven and fourteen days post wound creation, the wound sizes in the UP-treated group were significantly decreased, whereas those in the control and UM-treated groups were not significantly different as they were not completely healed (Figure 3B,C). Masson’s trichrome staining was done to investigate wound development in tissue samples of wounded skin. UP powder treatment resulted in more granulation tissue formation and collagen deposition than other treatments (Figure 3D). These results indicate that UP accelerates skin wound healing by enhancing collagen synthesis during the remodeling phase of the wound healing process.

### 3.5. Effect of U. parvifolia on Skin Wound Healing in Hairless Mice by Regulating MMP and TGF-β1 Expression

**Figure 4:**
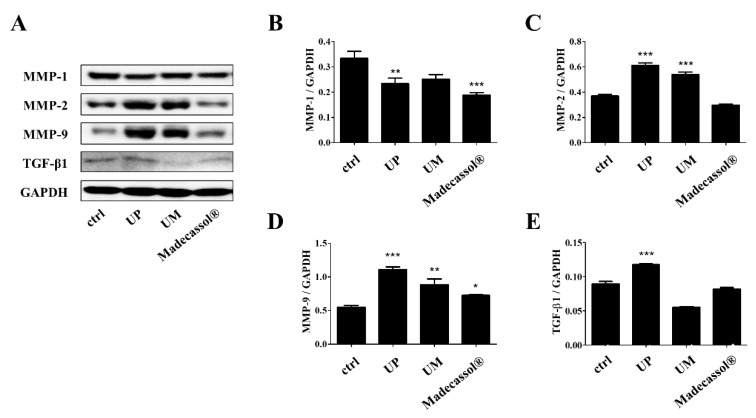
Effects of the root bark of *U. parvifolia* on the expression of MMPs and TGF-β1 in mouse dorsal skin tissue. (**A**) Western blot analyses of wounded skin showed the expression of (**B**) MMP-1, (**C**) MMP-2, (**D**) MMP-9, and (**E**) TGF-β1 on day 14 post-wounding. The values are shown as mean ± SEM (*n* = 7). * *p* < 0.05, ** *p* < 0.01, and *** *p* < 0.001 vs. the control group.

We explored the expression levels of MMP-1, -2, -9, and TGF-β1 in the mice on day 14 of UP treatment (Figure 4A). As shown in Figure 4B–E, UP treatment significantly decreased the protein expression of MMP-1 (UP: 29.81%, UM: 22.56% Madecassol^®^: 43.51%). On the contrary, the expression of MMP-2 or -9 was significantly upregulated in the UP-treated group compared with the Madecassol^®^-treated group (MMP-2: UP; 65.90%, UM; 46.18% Madecassol^®^; −19.36%, MMP-9: UP; 101.12%, UM; 60.27% Madecassol^®^; 31.51%). TGF-β1 levels were also increased in the UP-treated groups (UP: 31.81%, UM: −37.90% Madecassol^®^: −8.02%). These results indicate that UP can accelerate wound healing by enhancing the expression of MMP-2 and -9 and increasing TGF-β1 levels.

## 4. Discussion

Ulmus species have been widely used in Korean traditional medicine because of their anti-inflammatory and antimicrobial properties. Bioactive components, such as sesquiterpenoids, triterpenoids, flavonoids, coumarins, and lignans, are mainly present in this species [16]. It has been reported that UP has analgesic and anti-inflammatory effects [12,14]; however, its role in skin wound healing has not been reported. Therefore, in the present study, we demonstrated for the first time the skin wound healing effect of the root bark of UP in SKH-1 hairless mice.

As the population over 60 years of age grows, the burdens of nonhealing cutaneous wounds, such as pressure ulcers and diabetic foot ulcers, are increasing [17]. Cutaneous wounds are particularly hard to heal in aging, so it is necessary to develop effective treatments to heal wounds in aged skin.

For treatment of large-area wound injuries, such as pressure ulcers or rough or hard surfaces, a powder form of treatment is required and easy to apply. Treatment with powder in the wound area absorbs more wound exudate, forming a crust that prevents overdrying, and seals the wound from bacteria. It can modulate maintenance of moisture balance in the wound bed and also reduce the lingering of malodor compared to ointment application. [18]. In our study, it was observed that the wound closure and dermal regeneration effects of the 200 mesh root bark powder of UP were similar to those of Madecassol^®^, a commercially available wound healing ointment [19]. When the angle of repose and size of the particle were smaller, the solubility and water-retaining capacity of the powder were increased [20]. When the particle size of the UP powder was small, e.g., 200 mesh, it was able to hold more water than the UP powder with a larger size of particle (Figure S1). Interestingly, UP powder itself, instead of UP extract, has been used for many years in Korean traditional medicine. It is possible that the small particle size of UP powder itself may allow it to quickly absorb inflammatory exudate more than when presented in the form of a UP extract.

Maintaining hemostasis of collagen in the skin is a very important issue in skin rejuvenation and integrity for the wound matrix. It is also essential for re-epithelization and cell–cell and cell–matrix interactions. Deposition of collagen is important in wound healing and the development of wound strength [21]. The remodeling of collagenous proteins during wound healing can be influenced by proteolytic activities in the extracellular matrix by the MMPs. In our study, treatment of UP samples with different particle sizes showed that the finest UP powder (200 mesh) significantly decreased wound size in treated animals and also increased the collagen level in the dorsal skin.

During normal tissue remodeling and morphogenesis, MMPs play a crucial role in all stages of wound healing by modifying the wound matrix [22]. MMPs regulate cell–cell and cell–matrix signaling through the release of cytokines and growth factors sequestered in the ECM. Previously, it was shown that cytokines and hormones modulated MMP expression in skin tissues and could regulate inflammation and ECM on skin tissues [23]. In our study, expression of MMP-1 was downregulated by treatment of UP, similar to the positive control group. The loss of ECM may trigger MMP-1 expression in basal keratinocytes, thereby promoting migration, but keratinocytes downregulate the expression of MMP-1 in the final stage of tissue remodeling [24]. Furthermore, UP treatment upregulated MMP-2 and -9 expressions even more than those of Madecassol^®^ treatment. Particularly, overexpressions of matrix metalloproteinases 2 and 9 impair the remodeling and re-epithelization phases in wound-damaged models. [25]. Downregulation of MMP-2 and -9 expressions in the wounds increased keratinocyte migration during wound closure. MMP-9 knockout mice delay wound re-epithelialization and inhibit cell proliferation through Smad2 signaling in delaying corneal wound healing [26,27]. Therefore, the potential of MMPs and their inhibitors could be as therapeutic agents in treating wounds during distinct phases of the wound healing.

Keratinocyte migration and fibroblast migration during the re-epithelization phase are important processes in mammalian skin healing. Keratinocytes are the predominant cell type in the epidermis and are responsible for the epithelialization phase of skin wound healing. During epithelialization, keratinocytes proliferate and migrate to the wound site. These processes help ameliorate the disruption of the skin barrier [28]. Impaired keratinocyte migration results in poor wound healing, leading to a chronic wound [29]. Therefore, regulation of keratinocyte migration by UP treatment may ameliorate wound lesions via regulating expression of MMPs.

TGF-β is a family of growth factors that play an essential role in wound healing by regulating the inflammatory response, keratinocyte proliferation and migration, angiogenesis, collagen synthesis, and ECM remodeling. Lower TGF-β expression was studied in skin of a human diabetic foot ulcer [30]. Our results suggest that the potential efficacy of wound healing by UP seems to be due to stimulation of keratinocyte migration directly or TGF-β expression in the wound lesion.

Previous investigations conducted with the leaves of UP have demonstrated that it contains flavonol glycosides in its leaves [31]. Phytochemical constituents in the barks of *U*. *parvifolia* have resulted in the isolation of sterols, sterol glucoside, and a catechin glycoside [13]. In particular, catechin derivatives are among the major components in UP root bark and have a regulating cell migration effect (data not shown). Nevertheless, the scope of these claims is limited to the effect of the powder only. A future study with a major compound in the UP root bark could be studied. However, our study is a step forward in adding ethnopharmacological validation to the use of UP powder in wound healing cases.

## 5. Conclusions

For the first time, we discovered that the root bark powder of *U. parvifolia* could accelerate wound healing and that the mechanism might involve the upregulation of the expression of MMPs and TGF-β. Therefore, root bark powder of *U. parvifolia* can be a potential candidate in treating cutaneous wound damages. A further, precise mechanism study on UP in skin cells and the effects of its main compound should be done.

## Figures and Tables

**Table 1:** The angle of repose of *Ulmus parvifolia* (UP) root bark powder depending on particle size.

Mesh	Particle Size (μm)	Angle of Repose (θ)
20	355–850	57.2 ± 0.8
50	150	52.9 ± 0.7
100	75–150	51.9 ± 1.6
200	≤75	49.8 ± 1.1

## Supplementary Materials

The following are available online at https://www.mdpi.com/2077-0383/9/1/59/s1, Figure S1: Effect of particle size and soaking time on the water-holding capacity of different sized UP particles.
