# Supplementary material for: Ulmus parvifolia Accelerates Skin Wound Healing by Regulating the Expression of MMPs and TGF-β
Source: J Clin Med. 2019 Dec 26;9(1):59. doi: 10.3390/jcm9010059 (PMC7019489; doi:10.3390/jcm9010059)
Supplement: Supplementary file 1 [file jcm-09-00059-s001.pdf]

International Journal of
Molecular Sciences

MDPI

Supplementary:

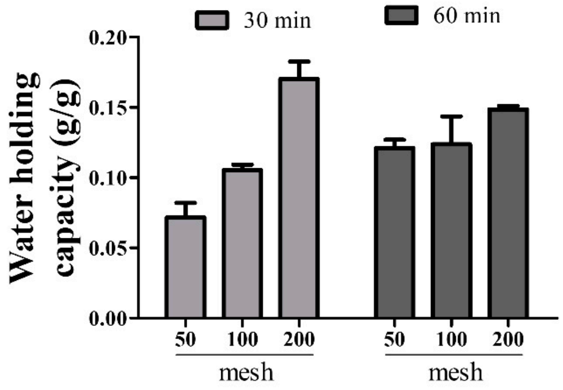

Figure S1. Effect of particle size and soaking time on the water holding capacity of different sized UP particles. This parameter was determined using the method of Zhao (2009)
